# The motivational basis of third-party punishment in children

**DOI:** 10.1371/journal.pone.0241919

**Published:** 2020-11-09

**Authors:** Mathias Twardawski, Benjamin E. Hilbig

**Affiliations:** 1 Social Psychology Lab, Department of Psychology, Ludwig-Maximilians-Universität München, Munich, Germany; 2 Cognitive Psychology Lab, Department of Psychology, University of Koblenz-Landau, Landau, Germany; Middlesex University, UNITED KINGDOM

## Abstract

People willingly accept personal costs to sanction norm violations even if they are not personally affected by the wrongdoing and even if their sanctioning yields no immediate benefits—a behavior known as third-party punishment. A notable body of literature suggests that this behavior is primarily driven by retribution (i.e., evening out the harm caused), rather than by the utilitarian motives of special prevention (i.e., preventing recidivism), or general prevention (i.e., preventing imitation). This has led to the conclusion that laypeople are “retributivists” in general. More recent evidence, however, raises doubts about the ubiquity of retributivism, showing that punishment is driven by multiple motives. The present research adds to this debate by investigating the motives underlying punishment in children around age 10. Specifically, we investigate children’s (*N* = 238) punishment motives in an economic game paradigm, isolating punishment motives by experimentally manipulating the extent to which the offender and a bystander learn about the punishment. This offers the possibility to examine whether (and to what extent) children engage in punishment even when it is devoid of any preventive effects. Results show that children’s punishment is motivated by retributive, special preventive, and general preventive purposes. These results point to a clear need for further theory specification on the motivational basis of punishment in humans and provide practical implications for the treatment of child misbehavior.

## Introduction

People willingly sacrifice their own resources (e.g., time or money) to sanction norm violations across a wide range of societies and contexts [1]. Strikingly, many individuals accept some personal costs to punish offenders even if they are not affected by the wrongdoing, and even if their sanctioning does not yield any immediate benefits [2]. In social psychology, behavioral economics, and related disciplines, this behavior is referred to as *third-party punishment* [3].

For over a decade, researchers have sought to understand the underlying motives driving third-party punishment [4–6], focusing on two main motives: *utilitarianism* and *retribution*. According to a utilitarian approach, punishment is an instrument to decrease norm violations and increase people’s compliance with social norms [1, 3]. Thus, punishment behavior is primarily forward-oriented and aims to prevent further misconduct, either by perpetrators themselves (termed *special prevention*) or by unrelated observers (termed *general prevention*) [5, 7]. By contrast, according to a retributive approach, punishment is intended to rebalance the moral wrong caused by the offense and seeks to pay back transgressors for their misbehavior [6, 8, 9]. Thus, punishment behavior is primarily backward-oriented and concerned with the harm caused rather than future actions [6]. Therefore, a retributive punishment ought to “fit the crime” [9] and, hence, retribution is also referred to as “the just deserts” theory [10].

A large body of research has analyzed people’s punishment decisions to understand whether there is a predominant objective underlying such behavior [5, 6, 9, 11]. For example, in this research, participants were provided with descriptions of crimes in which aspects that are relevant to retributive punishment (e.g., the magnitude of harm that has been done by the criminal) and aspects that are relevant to utilitarian punishment (e.g., the probability of recidivism or the public awareness of the crime and the sentencing) were varied. Results revealed that only crime aspects associated with retribution had an impact on participants’ punishment behavior, suggesting that individuals’ punishment is predominantly driven by retribution rather than by special or general preventive concerns [11]. Given that this parallels ample prior research investigating people’s punishment motives [6, 9, 12], scholars reached the apparent consensus that laypeople’s punishment is mostly driven by retribution [13].

However, more recent research suggests that the ubiquity of this “people as retributivists” position may, at least partially, be limited to certain methodological aspects of the research [14, 15]. Similarly, experiments studying punishment behavior in economic games revealed that punishment behavior is motivated by both retribution and special prevention [16, 17]. Of particular relevance for the research at hand, scholars isolated people’s punishment motives in a third-party punishment paradigm by manipulating the degree to which the punishment was communicated to the offenders and thus the extent to which it could entail any special preventive effects at all. As a core result, people were more willing to engage in punishment if it potentially facilitated special prevention, suggesting that punishment is not motivated by retribution alone [16]. Thus, it may at least be an oversimplification to conclude that individuals are strict retributivists in general.

The present research contributes to the debate on the motivational basis of punishment by studying punishment motives from a developmental perspective. Indeed, there is considerable evidence showing that children themselves engage in third-party punishment. More specifically, from early on, children express a general preference for equality [18], engage in punishment when they themselves have been harmed [19], and are willing to intervene and correct behavior that harms others [20]. In fact, third-party punishment has already been observed in children around age 6 [21, 22]. More recent research further investigated children’s understanding of the goals punishment intends to achieve [23]. In this work, children between the ages of 5 to 8 indicated whether they expected offenders to reoffend (i.e., special prevention) and observers to imitate the misbehavior (i.e., general prevention) in a context in which misbehavior leads to punishment vs. one without punishment. Results revealed that children expect a special preventive effect of punishment. However, findings regarding children’s understanding of general prevention were rather inconclusive, suggesting a delay in children’s understanding of the expressive function of punishment (i.e., sending a normative message to others through punishment) that does not emerge before age 8.

Although this research suggests that children’s punishment could be driven by both retribution and utilitarianism (i.e., at least by special prevention), evidence of the motives underlying actual third-party punishment behavior in children is relatively scarce. That is, to the best of our knowledge, only one study examined the principles underlying punishment in children thus far [24]. In this study, the developmental trajectories of retributive thinking were investigated by analyzing the extent to which children at different ages distributed unpopular tasks following equality vs. merit principles (with the latter reflecting a just deserts notion). It has been found that retributive thinking (vs. equality) in punishment only emerges (and aligns with adults’ behavior) by age 8–10. However, this research did not consider any other potential motives underlying children’s punishment (e.g., special or general prevention) and is therefore only partially informative for the question what drives punishment in children. Certainly, studying punishment motives in children still promises to contribute to further refining theories on the motivational basis of people’s punishment.

Beyond the goal of theory specification, investigating punishment motives in children is also insightful from an applied perspective. Children frequently experience misbehavior and punishment (as either a perpetrator, a victim, or an observer), for instance in school [25]. Such incidents are a main stressor for children [26, 27] that frequently lead to conflicts (e.g., with parents or teacher) that may have wide-ranging long-term consequences [28]. Such conflicts may arise due to differences between the expected (i.e., derived from one’s own perspective on punishment and its goals) and actually pursued objectives of punishment by authorities (e.g., teachers or parents). Indeed, recent research has shown that teachers intend to achieve preventive goals (rather than retribution) in various situations of student misbehavior [29]. The present research investigates whether children themselves also consider punishment a legitimate means to prevent future misbehavior and, thus, may reveal inherent differences between children’s and teacher’s perspectives on the purposes of punishment. Importantly, any such differences may decrease children’s compliance to rules and norms [30]. Therefore, it is of immediate applied relevance to understand children’s perspective on the treatment of misbehavior (e.g., for schools).

In summary, the present research adds to a relatively scarce literature on the motivational basis of punishment in children by directly investigating children’s motives to engage in third-party punishment themselves. In particular, we were interested in the extent to which punishment of children around age 10 is driven by retribution (i.e., evening out the harm caused), special prevention (i.e., preventing recidivism of the offender), and general prevention (i.e., preventing imitation of others). Studying children at this age is particularly suitable for the present research question, given that retributive thinking in children’s punishment has not been systematically observed for children younger than age 8 [24]. Furthermore, children only seem to comprehend the general preventive nature of punishment by that age [23]. Additionally, informal pretesting of the materials used in the present research revealed that these posed challenges to children younger than age 9.

To avoid well-documented biases, we applied a paradigm that does not require children to introspect on the motives they might have when punishing an offender, given that such approaches entail biased results even for adults [11, 16]. Instead, an approach from behavioral economics [31] was chosen, that is, an adaptation of the economic game sketched above that was recently introduced to study punishment motives of adults [16, 32]. For the present research, we modified this game to (a) make it comprehensible for children and (b) to differentiate between special prevention and general prevention as two distinct motives for punishment [5, 7, 14, 29, 33]. In this paradigm, participants observed another person (the offender) maximizing their own payoff (i.e., coins in a game) by acting unfairly towards another person. Participants then had the opportunity to reduce the offender’s payoff by investing resources of their own. To strictly isolate the unique effects of different punishment motives, it was experimentally manipulated whether the offender (special prevention), a bystander (general prevention), or nobody (retribution) learned about the punishment.

We report how we determined our sample size, all data exclusions (if any), all manipulations, and all measures [34]. All materials, the data, and analyses scripts are available online at the Open Science Framework and can be accessed via the following link: https://osf.io/u296x/

## Methods

### Ethics statement

The experiment reported herein was conducted in full accordance with the Ethical Guidelines of the German Association of Psychologists (DGPs) and the American Psychological Association’s (APA) Ethical Principles in the Conduct of Research with Human Participants. Ethics approval was given by the Ministry of Education, Science, Youth and Cultural Affairs Rhineland-Palatinate, as this research was conducted in German public schools. Informed (written) consent was provided by parents or legal guardians of the participants (as they were underage).

### Design and procedure

Data collection took place in three German public schools. On arrival in the classroom, children provided the experimenters with the informed consent form signed by their parents or legal guardians. Then, children were seated individually in front of computers. Two experimenters welcomed the class and gave them comprehensive verbal instructions on the subsequent task (using an example), which was a third-party punishment game. Children (always in the role of the punisher) were asked to imagine interacting with three other children: Child A (i.e., the distributor), Child B (i.e., the receiver), and Child C (i.e., the bystander). They were ask to imagine that they do not know the other children they interact with, nor would they ever meet them given that the interaction takes place in a virtual chatroom. There was also a fifth person in the chatroom whom we labeled “group manager.” This person’s function was merely to structure the interaction in the chatroom, reminding the participating children of the procedure of the task. Moreover, children learned that their answers would remain completely anonymous throughout the experiment, that is, they would never be asked to indicate their names to anyone in the chatroom. Finally, children were told that they were going to work on four rounds of this task, with changing individuals in the other roles.

**Fig 1:**
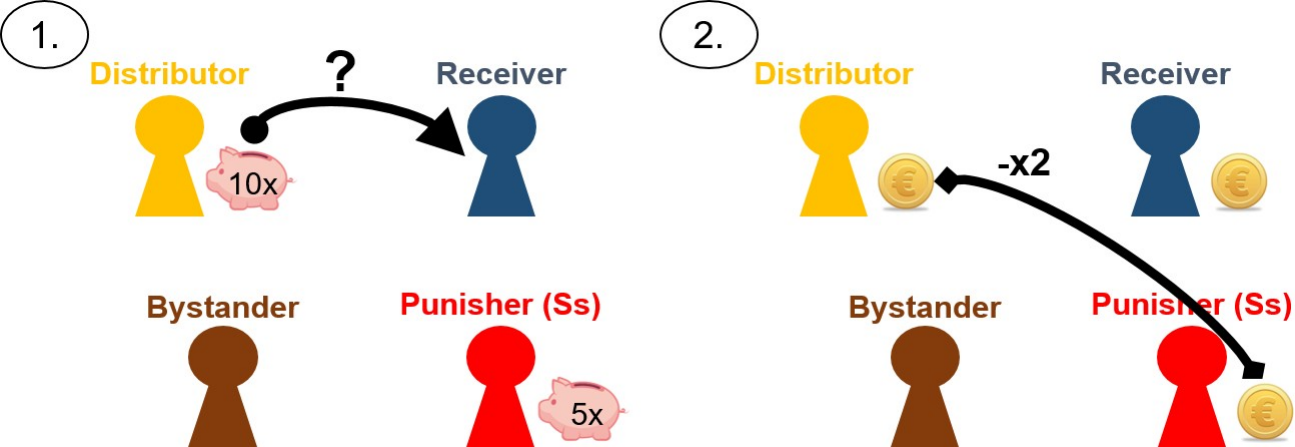
Overall structure of the economic game. Each round consisted of two steps. Initially, the distributor started with 10 piggy banks and the punisher started with five piggy banks. Each of these piggy banks contained a random number of valuable coins. (1) In step 1, the distributor decided how many of their own piggy banks should be given to the receiver. (2) In step 2, the punisher opened all piggy banks and counted the number of coins. Subsequently, the punisher had the power to reduce the distributor’s payoff by investing own coins; each coin spent resulted in a reduction of two coins for the distributor.

As depicted in Fig 1, each round consisted of two steps with the following rules: Initially, the distributor received 10 piggy banks and the punisher received five piggy banks. Each of these piggy banks contained a random number of valuable coins. In step 1, the distributor decided how many of their own piggy banks to give to the receiver. In step 2, the punisher privately opened all piggy banks and counted the number of coins each child (i.e., the punisher, the distributor, and the receiver) had. Importantly, participants were always confronted with an unfair distribution at this point. Subsequently, the punisher had the opportunity to reduce the distributor’s payoff by investing coins of their own; each coin invested by the punisher resulted in a reduction of two coins for the distributor. After the punisher’s decision, the round was over and all children in the chatroom received their final payoffs. The receiver and the bystander remained passive throughout.

Importantly, the game was constructed in a way that the other players (i.e., the distributor, the receiver, and the bystander) would not learn about the punishment enacted by the participant unless they received an explicit information about it. That is, final payoffs of all players (after step 2 of the interaction) were co-determined by (a) the (unequal) distribution of coins in the piggy banks and (b) the punishment enacted. Thus, without explicit information on the punishment, its presence and extent remain entirely unclear. For example, distributors ending up with four coins (the lowest amount this role could end up with) cannot tell whether they were unlucky in the first place (i.e., receiving several empty piggy banks) or whether they were punished in step 2. Consequently, punishment can only be applied for preventive purposes (e.g., to teach the distributor a lesson) if the punisher’s decision is explicitly communicated to others (e.g., the distributor).

**Fig 2:**
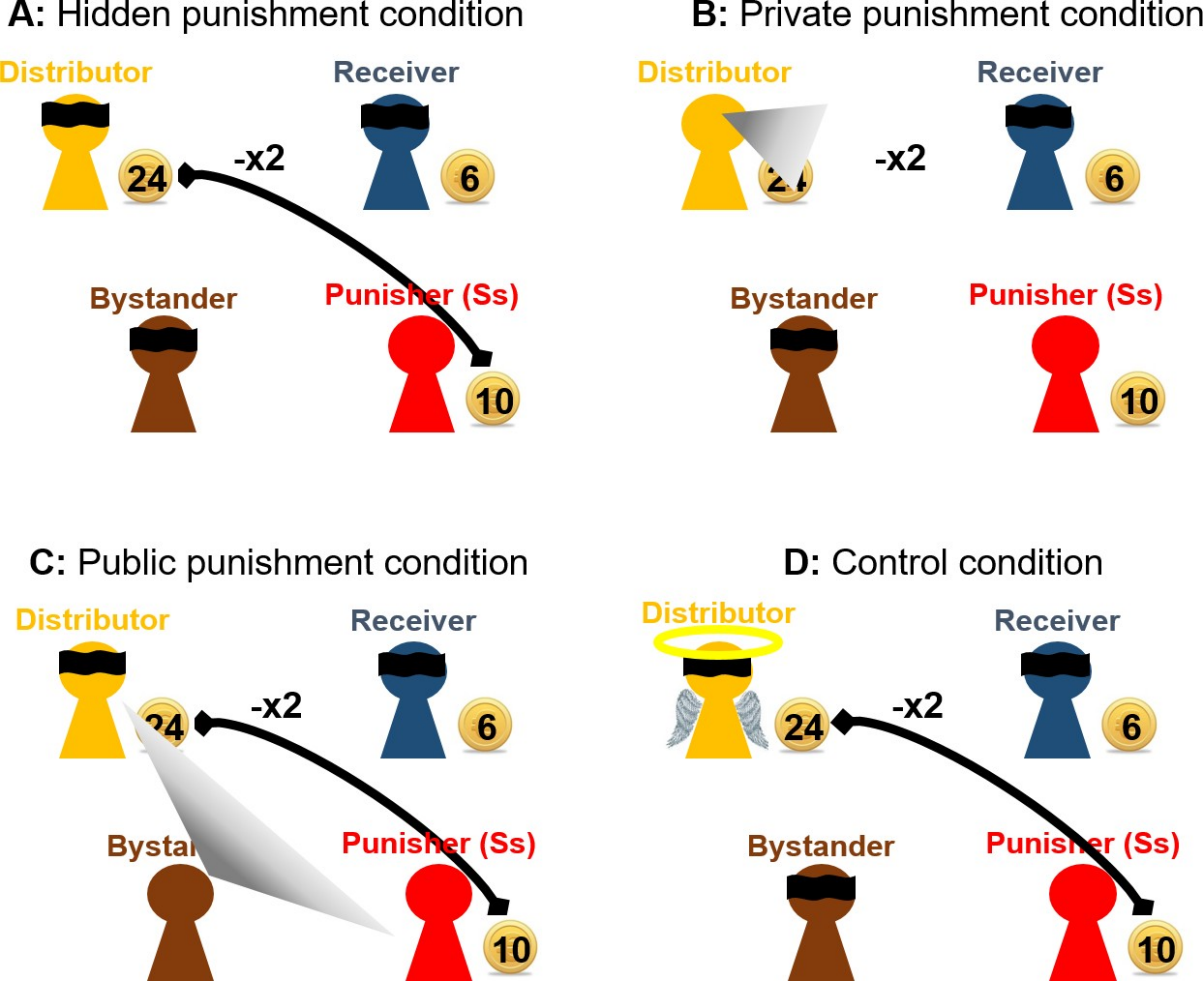
Experimental design. To isolate the unique effects of punishment motives (retribution vs. special prevention vs. general prevention) on the punisher’s decision, the distributor’s and bystander’s knowledge of whether and to what extent punishment had occurred was manipulated: (A) In the *hidden punishment condition*, neither the distributor nor the bystander learned about the punishment. (B) In the *private punishment condition*, the distributor (but not the bystander) learned about the punishment. (C) In the *public punishment condition*, the bystander (but not the distributor) learned about the punishment. (D) In the *control condition*, the distributor acted fairly towards the receiver (i.e., giving half of their piggy banks to the receiver) but still ended up with more coins than the receiver due to luck. Punishment in this condition may be interpreted as a decision to reinforce equality (rather than actually “punish” the distributor for a norm violation).

Crucially, we manipulated the distributor’s and the bystander’s knowledge of whether and to what extent the punisher decided to reduce the distributor’s payoff across four experimental conditions to be able to isolate the unique effects of punishment motives (retribution vs. special prevention vs. general prevention) on the punisher’s decision. Note that the group manager and the receiver were never informed about the children’s punishment decisions in any of the conditions. The four conditions are summarized in Fig 2 and will be described in detail below.

In the *hidden punishment condition* (see Fig 2A), neither the distributor nor the bystander were informed about whether and to what extent punishment had occurred. Given that punishment was not communicated to the distributor or the bystander in this condition, it could not prevent future misbehavior and, therefore, punishment in this condition can only be retributive (i.e., it is not motivated by preventing future misbehavior). In the *private punishment condition* (see Fig 2B), the distributor was informed whether and to what extent punishment had occurred. Thus, the distributor learned whether their own behavior is seen as legitimate and goes unpunished or not (i.e., adding special prevention as a punishment motive to retribution). In the *public punishment condition* (see Fig 2C), the bystander was informed whether and to what extent punishment had occurred. Thus, the bystander learned whether the distributor’s behavior shown in step 1 is seen as legitimate and goes unpunished or not (i.e., adding general prevention as a punishment motive to retribution). In the fourth condition, actual punishment motives were separated from other payoff-based motives (e.g., inequality aversion or spite), as these have been shown to affect children’s behavior [18, 35, 36]. In this *control condition* (see Fig 2D), the distributor acted fairly towards the receiver (i.e., giving half of the 10 piggy banks to the receiver) but still ended up with more coins than the receiver due to luck (i.e., the degree of inequality was similar to the other conditions). Therefore, the distributor’s behavior should not be interpreted as an offense requiring a punishment reaction by the punisher, but the punisher could nonetheless act to reinstate equality. The decision made in this condition was neither communicated to the distributor nor to the bystander and could thus be ascribed to purely payoff-based motives.

To reduce complexity for the children, they only participated in the role of the punisher. Thus, the interactions (including the punishment decision by the participant) in the chatroom were only hypothetical. Children were informed about this to avoid deception of participants [37, 38]. However, children were urged to imagine as best they can that this is a real interaction. To further reduce complexity, the differences between conditions were explicitly explained to the participating children, making sure that they knew that, for example, punishment in the hidden condition could not be used to teach the distributor a lesson. Moreover, all participating children started with the control condition, followed by the hidden condition (i.e., two rounds with the same rules but different behavior of the distributor). After explaining the changes in the rules (i.e., other children in the chatroom will learn about the punisher’s decision), children worked on the private and, finally, on the public condition. Furthermore, the distributor divided the piggy banks fairly in the control condition (i.e., giving half of the 10 piggy banks to the receiver), whereas the distributor acted very unfairly in all other conditions (i.e., giving only two of the 10 piggy banks to the receiver). Importantly, although the number of coins in the piggy banks was unknown by the time the distributor divided them, keeping more than half of the banks can arguably be interpreted as unfair. Indeed, there is substantial literature on both adults [3] and children [39], showing that individuals expect a fair distribution of resources in economic games and that behavior violating this expectation is perceived as a norm violation. Finally, to once more reduce complexity for the children, the distribution of coins after step 1 (i.e., after the decision made by the distributor) was consistent across the four conditions: the distributor always had 24 coins, the receiver always had six coins, and the punisher always had 10 coins. Thus, participating children were always provided with 10 coins to reduce the distributor’s payoff.

Note that we decided not to add comprehension check items within the study materials, given that data collection took place in classrooms and it was deemed crucial to avoid an atmosphere of testing (i.e., children thinking that there are correct and incorrect answers). Rather, we wanted to establish a relaxed and open atmosphere in which children were most likely to indicate their true opinions and to raise their hands whenever they needed assistance in understanding the tasks. Note, however, that this very rarely happened, suggesting the success of the substantial effort we put into ensuring the comprehensibility of the materials, including informal pretesting of the written and verbal instructions with children. That is, before collecting data in schools, we pretested our paradigm and instructions with six children between the age of 7 and 11 by applying the thinking-out-loud technique to test for comprehensibility and to further refine our materials in several rounds [40]. Furthermore, we increased comprehensibility for the children during our experiment by carefully explaining the procedures using concrete examples in the beginning, by verbally assuring that children understood the procedures during the examples (e.g., “Who’s turn is next, what do you think?”), and by leaving time and space for questions after the examples both in plenum and in private.

Following the third-party punishment game, children provided demographic information and then reached a stop sign asking them to wait until everyone else had finished this part of the experiment. Subsequently, they worked on an unrelated task. At the end of the experiment, children indicated the extent to which they experienced the tasks as difficult and enjoyable, before they were thanked and verbally debriefed.

### Sample

To calculate the required sample size, we conducted an a priori power analysis for a repeated measures ANOVA using G*Power [41, 42]. Specifically, we aimed to detect a small to medium-sized effect of *f* = .15 in a repeated measures ANOVA (number of groups = 1, number of measurements = 4 for the four conditions) with high power of 1-β = .90, given a conventional α = .05, and nonsphericity correction ε = 1. This resulted in a required sample size of *N* = 213 participants. Furthermore, a pragmatic approach was utilized before starting data collection, given that we collaborated with schools for this project. This prevented us from ceasing data collection exactly upon reaching the required sample size. Therefore, we planned to collect data in three schools and to recruit further schools for participation if we did not reach the threshold of approximately 215 participating children.

We collected data in the fifth and sixth grade of three public German schools. After 12 sessions, *N* = 238 children had participated in the experiment. Around 45% of participants (i.e., *n* = 106) were female, most children’s mother language was German (92%), and ages ranged between 9 and 12 (*M* = 10.46, *SD* = 0.61; one child did not indicate their age).

## Results

**Fig 3:**
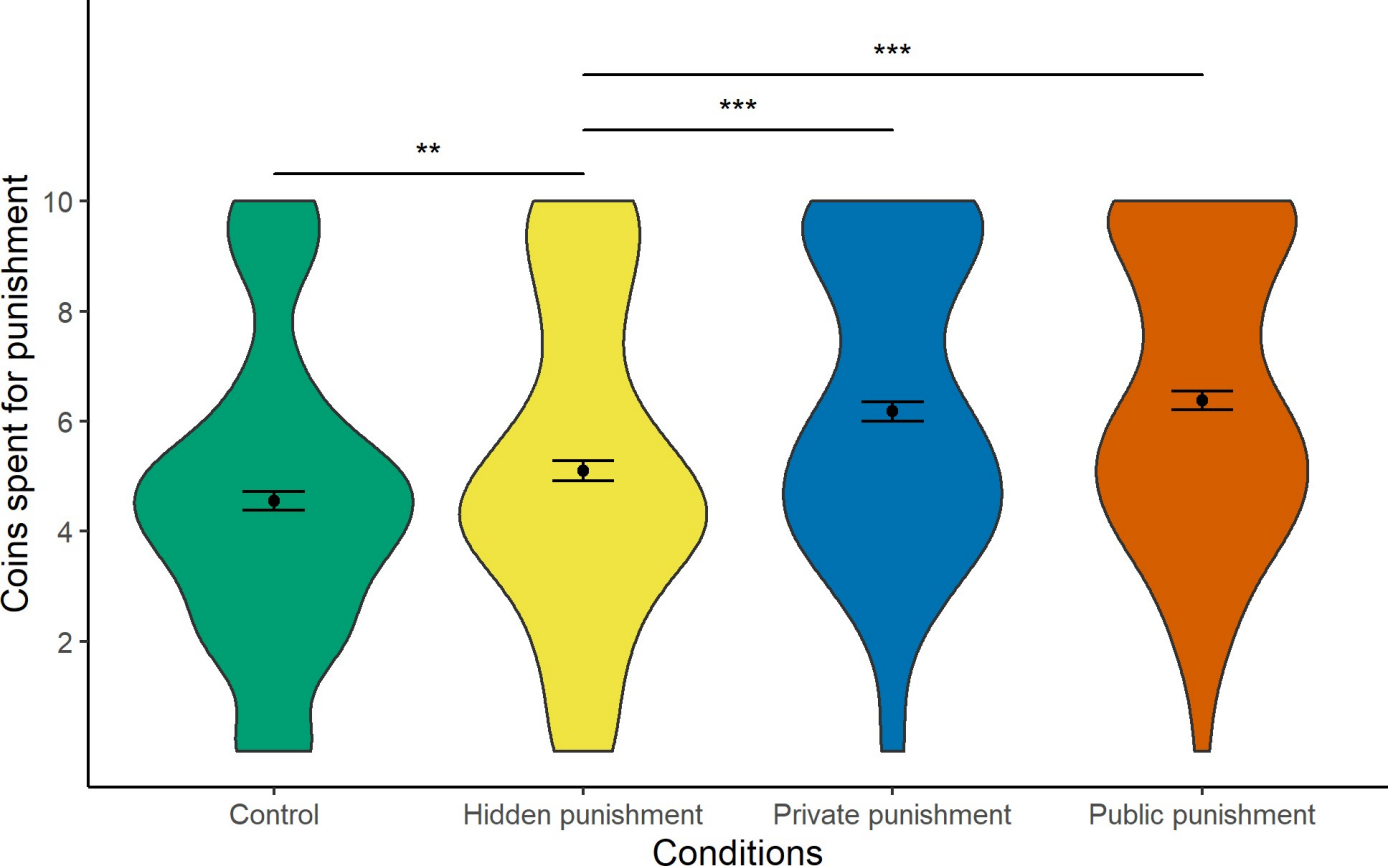
Coins spent for punishment in the four conditions. Differences between the control condition and the hidden condition can be interpreted as a proxy for children’s willingness to sacrifice own resources for the sake of retribution. Differences between the hidden condition and the private condition can be interpreted as a proxy for children’s willingness to sacrifice own resources for the sake of special prevention. Differences between the hidden condition and the public condition can be interpreted as a proxy for children’s willingness to sacrifice own resources for the sake of general prevention. Error bars represent one standard error of the mean.

Fig 3 displays the coins spent for punishment in the four conditions. As shown, the mean number of coins spent to reduce the distributor’s payoff was lowest in the control condition (*M* = 4.55, *SD* = 2.65). Punishment was slightly stronger in the hidden punishment condition (*M* = 5.10, *SD* = 2.82) and clearly strongest in the two preventive conditions private punishment (*M* = 6.18, *SD* = 2.69) and public punishment (*M* = 6.38, *SD* = 2.64), which turned out roughly comparable.

To test the unique effects of punishment motives on children’s punishment behavior, we conducted a repeated-measures ANOVA with the four experimental conditions being contrast-coded into three dummy variables (D1, D2, D3). D1 reflects the contrast for retribution, comparing the control condition (i.e., fair behavior of the distributor and an advantage in coins due to luck) with the hidden punishment condition (i.e., unfair behavior of the distributor with hidden punishment). A significant effect of D1 indicates a punishment beyond an intervention due to purely payoff-based motives such as inequality aversion and without the potential benefit of preventing future misbehavior. Thus, children’s willingness to sacrifice these additional coins is driven by retribution. D2 reflects the contrast for special prevention, comparing the hidden punishment condition (i.e., unfair behavior of the distributor with hidden punishment) with the private punishment condition (i.e., unfair behavior of the distributor with a punishment communicated to the distributor). A significant effect of D2 indicates a punishment beyond an intervention due to retributive purposes and is therefore motivated by special prevention (i.e., teaching the distributor a lesson). D3 reflects the contrast for general prevention, comparing the hidden punishment condition (i.e., unfair behavior of the distributor with hidden punishment) with the public punishment condition (i.e., unfair behavior of the distributor with a punishment communicated to the bystander). A significant effect of D3 indicates a punishment beyond an intervention due to retributive purposes and is therefore motivated by general prevention (i.e., communicating to the bystander that unfair behavior cannot go unpunished).

Importantly, there were significant differences in punishment across conditions, *F*(2.67, 632.78) = 41.97, *p* < .001, $\eta_{G}^{2}$ = .07 (note that given Mauchly’s test indicated that the assumption of sphericity was violated, a Greenhouse–Geisser corrected test is reported, ε = .89). Contrasts testing the unique effects of each motive on children’s punishment behavior revealed a significant effect of D1 that reflects differences between fair and unfair behavior with a non-communicated punishment, and thus, indicates a retributive punishment beyond an intervention due to purely payoff-based motives such as inequality aversion, *t*(237) = 2.96, *p* = .003, *d* = 0.19. Furthermore, there was a significant effect of D2, reflecting greater punishment in the condition allowing the punisher to teach the distributor a lesson than in the condition in which punishment was purely retributive, *t*(237) = 5.74, *p* < .001, *d* = .37. This indicates that children’s punishment was also largely motivated by special prevention. Likewise, D3 was also significant, reflecting greater punishment in the condition allowing the punisher to communicate a norm of fairness (in which misbehavior gets punished) to a bystander than in the condition in which punishment was purely retributive, *t*(237) = 6.15, *p* < .001, *d* = .40. This indicates that children’s punishment was also largely motivated by general prevention.

## Discussion

Most literature on laypeople’s punishment motives suggests that individuals’ punishment is best described as primarily retributive, rather than occurring for utilitarian purposes such as special prevention or general prevention [5, 6, 9]. More recent research, however, provides evidence against the ubiquity of retributive motives [16, 17]. The present research adds to the debate on the motivational basis of punishment by investigating punishment motives from a developmental perspective. In fact, although there is considerable evidence that children engage in third-party punishment from a young age [21, 22], the motives underlying this behavior in children has, so far, received little attention. Therefore, we examined the extent to which punishment behavior of children around age 10 is driven by retribution (i.e., to even out the harm caused), special prevention (i.e., to prevent future misconduct by the offender), or general prevention (i.e., to prevent future misconduct by others) [6, 9].

To this end, we adapted a third-party punishment game that has recently been introduced to examine adults’ punishment motives [16]. In the game, children observed another child accumulating payoffs by acting unfairly. Children then had the opportunity to punish, that is, to reduce the offender’s payoff by sacrificing own resources. To isolate the unique effects of the punishment motives, it was manipulated across experimental conditions whether the offender (i.e., important for special prevention), a bystander (i.e., important for general prevention), or nobody (i.e., retribution) learned about the punishment.

Results revealed that children’s third-party punishment is driven by both retributive and preventive purposes. More precisely, reflecting retributive motives in punishment, children were willing to reduce an offender’s payoffs exclusively for the sake of punishment and without the chance to prevent future misbehavior (as they knew that no one would learn about the punishment). Importantly, however, punishment was also motivated by special prevention and general prevention. That is, compared to a hidden punishment situation (i.e., nobody learns about the punishment), children were willing to sacrifice *additional* resources both when the offender learned about the punishment (i.e., special prevention), and when a bystander learned about the punishment (i.e., general prevention).

These results have several implications, both applied and theoretical. From an applied perspective, the present results bear implications for teachers (and parents) handling children’s misbehavior on a daily basis [25]. In school, student misbehavior is a main cause of conflicts, regularly leading to feelings of injustice in students [26, 27]. Knowing that children’s punishment is motivated by both retribution and utilitarianism (i.e., both purposes of punishment are generally supported by children) could provide teachers with more confidence in the treatment of child misbehavior, something many teachers feel they are lacking [43]. As such, teachers may want to frame their punishment as intending to achieve both retributive and preventive purposes. This result is particularly important given that differences between expected and actually pursued punishment objectives (of authorities) may actually decrease children’s compliance to rules and norms [30].

On a theoretical level, the present research adds to an ongoing debate on whether laypeople’s punishment is driven by retribution or utilitarianism [6, 9, 14]. Our findings suggest that, from a developmental perspective, humans are both retributivists and utilitarians. This contrast with earlier notions suggesting that third-party punishment is predominantly driven by retribution [5, 6, 9] and adds to more recent literature challenging the ubiquity of retribution. Of note, we adapted an economic game [16] that stems from research on adults’ punishment motives that provided evidence *against* the notion that people’s punishment is predominantly driven by retribution–something we now confirm in children. Nevertheless, the results that children around age 10 engage in third-party punishment for retributive, special preventive, and general preventive purposes calls for further theory specification and more empirical work on the motivational basis of punishment in humans [21, 22, 24].

That said, caution is required concerning the developmental claims that can be made from a sample of children around age 10. That is, although we are among the first directly investigating punishment motives in children, our work arguably only provides preliminary insights into the developmental trajectories of laypeople’s punishment motives. More precisely, given that children already engage in third-party punishment around age 6 [21, 22, 24], future research may study the motivational basis of punishment in younger children–or may even apply a longitudinal approach to examine the developmental origins of individuals’ punishment motives in more detail.

Furthermore, in the present research, we analyzed children’s *decisions* made under varying circumstances to isolate the motives of their punishment. However, research investigating children’s punishment may benefit from a deeper analysis of the psychological mechanisms underlying such behavior. For example, scholars could examine the emotional foundations of children’s punishment (and its goals). In fact, there is plenty of evidence relating people’s behavioral responses to moral violations (e.g., punishment) to various emotions such as anger, outrage, and moral disgust [44, 45]. Importantly, recent research suggests that retribution is rather concerned with the crime itself, whereas utilitarian motives (e.g., special prevention or general prevention) are more strongly linked to concerns about the offender or the community the offense occurred in [15]. Therefore, one could speculate whether different emotional responses to the perceived offense may be related to different punishment motives. For example, more “crime-focused” emotions such as anger about the misbehavior may be associated with a retributive punishment, whereas “other-focused” emotions such as compassion for the victim or pity for the offender may be associated with a utilitarian punishment (e.g., special prevention) [46, 47]. In sum, examining the emotional underpinnings alongside the motivational basis of children’s punishment may be an additional promising avenue for future research.

In addition to the constraints outlined above, there are also potential methodological limitations of the present research that warrant discussion. For example, the stronger punishment in the private punishment condition may to some extent also be driven by a retributive desire to inflict emotional harm (in addition to material costs) rather than achieving any future developments (i.e., special prevention) [16]. That is, punishers may arguably anticipate that the communication of punishment to the distributor could be an additional, non-material sanction. Therefore, it is possible that the present paradigm underestimated the extent to which retributive motives were the driver of third-party punishment in this condition. Importantly, however, this reasoning cannot account for the additional punishment assigned in the public punishment condition, simply because the offender remains anonymous (towards the bystander) and unaware of whether punishment even occurred. Nevertheless, future research may conceptually replicate our findings by applying other methods, such as information-search tasks [5, 6].

Furthermore, we applied a within-subjects design, which potentially may have resulted in demand characteristics (e.g., carry-over effects). Although within-subject designs bear notable advantages over between-subjects designs (e.g., in terms of statistical power), the issue of carry-over effects is particularly serious, as the order of conditions in our study was fixed. We opted for a fixed order to reduce complexity for the children and ensure comprehensiveness. Indeed, due to the fixed order of conditions, we were able to explain the changes made between conditions in plenum, rather than asking children to read and understand the instructions themselves. However, future research may replicate the present findings in a between-subjects design to circumvent potential demand characteristics.

Moreover, we did not include any comprehension check questions testing (and thus ensuring) children’s understanding of the rules of our paradigm. We did this to avoid that children think there were “correct” decisions that should be made, instead of making their own decisions (something we feared as data collection took place in classrooms). However, refraining from including such comprehension checks comes with the disadvantage that we cannot guarantee all children understood the changes between conditions and the consequences resulting from these changes. In fact, one could argue that some conditions (e.g., the private or public punishment conditions) contain higher comprehension demands as compared to other conditions (e.g., the hidden condition) and that condition differences (or the lack thereof) may be caused by these differences. Importantly, however, both in preparing the study materials (e.g., by informal pretesting) and during data collection (e.g., by carefully explaining the conditions), we put great effort into ensuring that all participating children understood the paradigm comprehensively. Indeed, the participating children themselves rated the tasks of the experiment as easily understandable (*M* = 4.38, *SD* = 0.86, *Md* = 5; on a scale from 1 to 5). Moreover, removing children scoring lower than 3 (i.e., the middle option) on this question (*n* = 7) did not have any impact on the results of the analyses. Hence, although we cannot rule out this potential confound conclusively with the present data, we are confident that most (if not all) children in our sample fully understood the rules of the paradigm and that the general results of the present research are unaffected by any comprehension problems.

In sum, the present research is among the first to investigate the motives underlying third-party punishment in children. We found that children’s punishment is not only motivated by retribution (i.e., to even out the harm caused), but also by special prevention (i.e., to prevent future misconduct by the offender) and general prevention (i.e., to prevent future misconduct by others). These results shed initial light on children’s punishment motives and thus, both contribute to an ongoing debate on the motivational basis of punishment in humans [6, 8, 13, 16] and provide applied implications for the treatment of child misbehavior (e.g., in school) [43].

## References

[1] Henrich J, McElreath R, Barr A, Ensminger J, Barrett C, Bolyanatz A, et al Costly punishment across human societies. Science (80-). 2006;312: 1767–1770. 10.1126/science.1127333 16794075

[2] Fehr E, Gächter S. Altruistic punishment in humans. Nature. 2002;415: 137–40. 10.1038/415137a 11805825

[3] Fehr E, Fischbacher U. Third-party punishment and social norms. Evol Hum Behav. 2004;25: 63–87. 10.1016/S1090-5138(04)00005-4

[4] Boyd R, Gintis H, Bowles S. Coordinated punishment of defectors sustains cooperation and can proliferate when rare. Science (80-). 2010;328: 617–620. 10.1126/science.1183665 20431013

[5] Keller LB, Oswald ME, Stucki I, Gollwitzer M. A closer look at an eye for an eye: Laypersons’ punishment decisions are primarily driven by retributive motives. Soc Justice Res. 2010;23: 99–116. 10.1007/s11211-010-0113-4

[6] Carlsmith KM, Darley JM, Robinson PH. Why do we punish? Deterrence and just deserts as motives for punishment. J Pers Soc Psychol. 2002;83: 284–299. 10.1037/0022-3514.83.2.284 12150228

[7] Rucker DD, Polifroni M, Tetlock PE, Scott AL. On the assignment of punishment: The impact of general-societal threat and the moderating role of severity. Personal Soc Psychol Bull. 2004;30: 673–684. 10.1177/0146167203262849 15155032

[8] Goodwin GP, Gromet DM. Punishment. Wiley Interdiscip Rev Cogn Sci. 2014;5: 561–572. 10.1002/wcs.1301 26308745

[9] Carlsmith KM. The roles of retribution and utility in determining punishment. J Exp Soc Psychol. 2006;42: 437–451. 10.1016/j.jesp.2005.06.007

[10] Darley JM, Carlsmith KM, Robinson PH. Incapacitation and just deserts as motives for punishment. Law Hum Behav. 2000;24: 659–683. 10.1023/a:1005552203727 11105478

[11] Carlsmith KM. On justifying punishment: The discrepancy between words and actions. Soc Justice Res. 2008;21: 119–137. 10.1007/s11211-008-0068-x

[12] Roberts J V, Gebotys RJ. The purposes of sentencing: Public support for competing aims. Behav Sci Law. 1989;7: 387–402. 10.1002/bsl.2370070308

[13] Carlsmith KM, Darley JM. Psychological aspects of retributive justice. Adv Exp Soc Psychol. 2008;40: 193–236. 10.1016/S0065-2601(07)00004-4

[14] Goodwin GP, Benforado A. Judging the goring ox: Retribution directed toward animals. Cogn Sci. 2015;39: 619–646. 10.1111/cogs.12175 25256421

[15] Twardawski M, Tang KTY, Hilbig BE. Is it all about retribution? The flexibility of punishment goals. Soc Justice Res. 2020;33: 195–218. 10.1007/s11211-020-00352-x

[16] Crockett MJ, Özdemir Y, Fehr E. The value of vengeance and the demand for deterrence. J Exp Psychol Gen. 2014;143: 2279–2286. 10.1037/xge0000018 25285429 PMC4242077

[17] Deutchman P, Bračič M, Raihani N, McAuliffe K. Punishment is strongly motivated by revenge and weakly motivated by inequity aversion. Evol Hum Behav. 2020;703: 135577 10.1016/j.evolhumbehav.2020.06.001

[18] Hamann K, Warneken F, Greenberg JR, Tomasello M. Collaboration encourages equal sharing in children but not in chimpanzees. Nature. 2011;476: 328–331. 10.1038/nature10278 21775985

[19] Robbins E, Rochat P. Emerging signs of strong reciprocity in human ontogeny. Front Psychol. 2011;2: 353 10.3389/fpsyg.2011.00353 22194730 PMC3242362

[20] Kenward B, Östh T. Enactment of third-party punishment by 4-year-olds. Front Psychol. 2012;3: 1–9. 10.3389/fpsyg.2012.00001 23162486 PMC3498893

[21] Jordan JJ, McAuliffe K, Warneken F. Development of in-group favoritism in children’s third-party punishment of selfishness. Proc Natl Acad Sci. 2014;111: 12710–12715. 10.1073/pnas.1402280111 25136086 PMC4156744

[22] McAuliffe K, Jordan JJ, Warneken F. Costly third-party punishment in young children. Cognition. 2015;134: 1–10. 10.1016/j.cognition.2014.08.013 25460374

[23] Bregant J, Shaw A, Kinzler KD. Intuitive jurisprudence: Early reasoning about the functions of punishment. J Empir Leg Stud. 2016;13: 693–717. 10.1111/jels.12130

[24] Smith CE, Warneken F. Children’s reasoning about distributive and retributive justice across development. Dev Psychol. 2016;52: 613–628. 10.1037/a0040069 26845506 PMC4808465

[25] Kulinna PH, Cothran DJ, Regualos R. Teachers’ reports of student misbehavior in physical education. Res Q Exerc Sport. 2006;77: 32–40. 10.1080/02701367.2006.10599329 16646350

[26] Fan RM, Chan SCN. Students’ perceptions of just and unjust experiences in school. Educ Child Psychol. 1999;16: 32–50.

[27] Israelashvili M. Situational determinants of school students’ feelings of injustice. Elem Sch Guid Couns. 1997;31: 283–292.

[28] Wald J, Losen DJ. Defining and redirecting a school-to-prison pipeline. New Dir Youth Dev. 2003; 9–15. 10.1002/yd.51 14635431

[29] Twardawski M, Hilbig BE, Thielmann I. Punishment goals in classroom interventions: An attributional approach. J Exp Psychol Appl. 2020;26: 61–72. 10.1037/xap0000223 30985157

[30] Mooijman M, van Dijk WW, van Dijk E, Ellemers N. On sanction-goal justifications: How and why deterrence justifications undermine rule compliance. J Pers Soc Psychol. 2017;112: 577–588. 10.1037/pspi0000084 27935728

[31] Gummerum M, Hanoch Y, Keller M. When child development meets economic game theory: An interdisciplinary approach to investigating social development. Hum Dev. 2008;51: 235–261. 10.1159/000151494

[32] Nadelhoffer T, Heshmati S, Kaplan D, Nichols S. Folk retributivism and the communication confound. Econ Philos. 2013;29: 235–261. 10.1017/S0266267113000217

[33] Tetlock PE. Social functionalist frameworks for judgment and choice: Intuitive politicians, theologians, and prosecutors. Psychol Rev. 2002;109: 451–471. 10.1037/0033-295x.109.3.451 12088240

[34] Simmons JP, Nelson LD, Simonsohn U. A 21 word solution. Dialogue Off Newsl Soc Personal Soc Psychol. 2012;26: 4–7.

[35] Lobue V, Nishida T, Chiong C, Deloache JS, Haidt J. When getting something good is bad: Even three-year-olds react to inequality. Soc Dev. 2011;20: 154–170. 10.1111/j.1467-9507.2009.00560.x

[36] Blake PR, McAuliffe K, Warneken F. The developmental origins of fairness: the knowledge–behavior gap. Trends Cogn Sci. 2014;18: 559–561. 10.1016/j.tics.2014.08.003 25175834

[37] Hertwig R, Ortmann A. Deception in social psychological experiments: Two misconceptions and a research agenda*. Soc Psychol Q. 2008;71: 222–227. 10.1177/019027250807100304

[38] Ortmann A, Hertwig R. The costs of deception: Evidence from psychology. Exp Econ. 2002;5: 111–131. 10.2139/ssrn.317861

[39] Fehr E, Bernhard H, Rockenbach B. Egalitarianism in young children. Nature. 2008;454: 1079–1083. 10.1038/nature07155 18756249

[40] Olson GM, Duffy SA, Mack RL. Thinking-out-loud as a method for studying 11 real-time comprehension processes. New methods Read Compr Res. 2018;253.

[41] Faul F, Erdfelder E, Lang A-G, Buchner A. G*Power 3: A flexible statistical power analysis program for the social, behavioral, and biomedical sciences. Behav Res Methods. 2007;39: 175–191. 10.3758/bf03193146 17695343

[42] Faul F, Erdfelder E, Buchner A, Lang A-G. Statistical power analyses using G*Power 3.1: Tests for correlation and regression analyses. Behav Res Methods. 2009;41: 1149–1160. 10.3758/BRM.41.4.1149 19897823

[43] Melnick SA, Meister DG. A comparison of beginning and experienced teachers’ concerns. Educ Res Q. 2008;31: 40–56. 10.1017/CBO9781107415324.004

[44] Haidt J. The moral emotions. In: Davidson RJ, Scherer KR, Goldsmith HH, editors. Handbook of affective sciences. Oxford: Oxford University Press; 2003 pp. 852–870.

[45] Molho C, Tybur JM, Güler E, Balliet D, Hofmann W. Disgust and anger relate to different aggressive responses to moral violations. Psychol Sci. 2017;28: 609–619. 10.1177/0956797617692000 28485700 PMC5426557

[46] Pfattheicher S, Sassenrath C, Keller J. Compassion magnifies third-party punishment. J Pers Soc Psychol. 2018 10.1007/s00402-009-0880-2 30945902

[47] Kogut T. The role of perspective taking and emotions in punishing identified and unidentified wrongdoers. Cogn Emot. 2011;25: 1491–1499. 10.1080/02699931.2010.547563 21432640

